# Virus-induced p38 MAPK activation facilitates viral infection

**DOI:** 10.7150/thno.50992

**Published:** 2020-10-30

**Authors:** Yuting Cheng, Fang Sun, Luyao Wang, Minjun Gao, Youli Xie, Yu Sun, Huan Liu, Yufeng Yuan, Wei Yi, Zan Huang, Huan Yan, Ke Peng, Yingliang Wu, Zhijian Cao

**Affiliations:** 1 State Key Laboratory of Virology and Modern Virology Research Center, College of Life Sciences, Wuhan University, Wuhan 430072, Hubei, China.; 2 Jiangsu Agri-animal Husbandry Vocational College, Jiangsu Key Laboratory for High-Tech Research and Development of Veterinary Biopharmaceuticals, Taizhou, 225300, China.; 3 Zhongnan Hospital of Wuhan University, Wuhan 430071, Wuhan 430072, Hubei, China.; 4 School of Medicine, Wuhan University of Science and Technology, Wuhan 430065, Hubei, China.; 5 Department of Neurosurgery, Renmin Hospital of Wuhan University, Wuhan 430060, Hubei, China.; 6 Wuhan Institute of Virology, Chinese Academy of Sciences, Wuhan 430071, Hubei, China.; 7 Hubei Province Engineering and Technology Research, Center for Fluorinated Pharmaceuticals, Wuhan University, Wuhan 430071, Hubei, China.

**Keywords:** P38 activation, TAB1, HCV, SFTSV, SARS-CoV-2

## Abstract

**Rationale:** Many viral infections are known to activate the p38 mitogen-activated protein kinase (MAPK) signaling pathway. However, the role of p38 activation in viral infection and the underlying mechanism remain unclear. The role of virus-hijacked p38 MAPK activation in viral infection was investigated in this study.

**Methods:** The correlation of hepatitis C virus (HCV) infection and p38 activation was studied in patient tissues and primary human hepatocytes (PHHs) by immunohistochemistry and western blotting. Coimmunoprecipitation, GST pulldown and confocal microscopy were used to investigate the interaction of p38α and the HCV core protein. *In vitro* kinase assays and mass spectrometry were used to analyze the phosphorylation of the HCV core protein. Plaque assays, quantitative real time PCR (qRT-PCR), western blotting, siRNA and CRISPR/Cas9 were used to determine the effect of p38 activation on viral replication.

**Results:** HCV infection was associated with p38 activation in clinical samples. HCV infection increased p38 phosphorylation by triggering the interaction of p38α and TGF-β activated kinase 1 (MAP3K7) binding protein 1 (TAB1). TAB1-mediated p38α activation facilitated HCV replication, and pharmaceutical inhibition of p38α activation by SB203580 suppressed HCV infection at the viral assembly step. Activated p38α interacted with the N-terminal region of the HCV core protein and subsequently phosphorylated the HCV core protein, which promoted HCV core protein oligomerization, an essential step for viral assembly. As expected, SB203580 or the HCV core protein N-terminal peptide (CN-peptide) disrupted the p38α-HCV core protein interaction, efficiently impaired HCV assembly and impeded normal HCV replication in both cultured cells and primary human hepatocytes. Similarly, severe fever with thrombocytopenia syndrome virus (SFTSV), herpes simplex virus type 1 (HSV-1) or severe acute respiratory syndrome coronavirus 2 (SARS-CoV-2) infection also activated p38 MAPK. Most importantly, pharmacological blockage of p38 activation by SB203580 effectively inhibited SFTSV, HSV-1 and SARS-CoV-2.

**Conclusion:** Our study shows that virus-hijacked p38 activation is a key event for viral replication and that pharmacological blockage of p38 activation is an antiviral strategy.

## Introduction

Viral infections remain a serious threat to human health. Viral diseases include the common cold, immunodeficiencies and cancers. For example, chronic hepatitis C virus (HCV) infection affects more than 71 million people worldwide (https://www.who.int/). Affected individuals have a high risk of developing cirrhosis within 20 years that ranges from 15% to 30%, which may ultimately cause fatal hepatocellular carcinoma 1. Although pangenotypic direct-acting antivirals (DAAs) effectively cure most persons with HCV infection, access to HCV treatment remains very limited due to the high cost. Furthermore, antiviral drug resistance has become an issue, and various resistance mutations against DAA treatment have been documented 2, 3. As another example, the tick-borne virus SFTSV (severe fever with thrombocytopenia syndrome virus) leads to severe fever with thrombocytopenia syndrome. Patients with SFTS have high fatality rate of 12%-50% 4. Although SFTSV infection is often transmitted via tick bites in East Asia, including China, Korea and Japan, there are still no specific anti-SFTSV drugs for use in the clinic. Herpes simplex virus type 1 (HSV-1) is a common human pathogen that causes severe diseases, including mucocutaneous lesions in the oral mucosa, encephalitis, meningitis, and blinding keratitis. Although there are many clinical drugs with effects against HSV-1, HSV infection remains a serious challenge because of viral resistance and side effects 5. Currently, the newly emerged virus SARS-CoV-2 is causing a global coronavirus disease 2019 (COVID-19) pandemic in 235 countries, areas or territories. As of Oct. 10^th^, the World Health Organization (WHO) had reported approximately 36,361,054 confirmed cases globally with 1,056,186 deaths. Moreover, the epidemic of COVID-19 is not well controlled because of the lack of specific antiviral drugs and vaccines. These results demonstrate the need for more mechanistic studies to provide novel strategies for the development of antiviral therapy.

P38 mitogen-activated protein kinases (MAPK) play crucial roles in signaling cascade responses to various cellular stimuli 6, including viral infections. Infections by many viruses, such as HCV 7, influenza virus 8, enterovirus 71 (EV71) 9, human immunodeficiency virus (HIV) 10, dengue virus (DENV) 11, and hepatitis B virus (HBV) 12, can activate p38 MAP kinases. There are four p38 kinase members in the mitogen-activated protein (MAP) family in mammals, p38α, p38β, p38γ and p38δ, which play crucial roles in many biological processes in response to extracellular stimuli that mediate a variety of biological processes, such as inflammation, cell proliferation, apoptosis and differentiation, aging and tumorigenesis 13-15. P38 MAPK is prototypically modulated by MAP3K, MAPKK, and MAPK in the classical pathway 16-18. Alternatively, p38α can also be autophosphorylated by interacting with TGF-β activated kinase 1 (MAP3K7)-binding protein 1 (TAB1) 19-21. In addition, p38 can be activated by the TCR-mediated intracellular tyrosine kinase Zap70 in T cells 22. Although multiple viruses have been shown to activate p38 MAPK, the underlying mechanisms have not been dissected. Furthermore, the pathophysiological role of p38 activation in viral infections remains controversial.

In this study, we focused on the clinical and biological importance of p38 MAPK in viral infections, and revealed a new mechanism by which HCV infection triggers TAB1-dependent p38 activation to phosphorylate the HCV core protein to promote HCV replication. Like HCV, the infection of SFTSV, HSV-1 or SARS-CoV-2 also induced p38 activation, and the p38 inhibitor SB203580 can affect the replication of SFTSV, HSV-1 and SARS-CoV-2. Therefore, p38 activation may be a key event in viral infections, and targeting p38 activation may be a promising strategy for combatting many viruses, including SFTSV or SARS-CoV-2, that cause potentially fatal infections.

## Materials and Methods

### Ethics Statement

Liver cancer samples from HCV positive or HCV negative patients and blood samples from HCV-infected or healthy donors were collected at Zhongnan Hospital of Wuhan University, China. The subjects or their family members voluntarily signed informed consent forms. The Zhongnan Hospital of the Wuhan University Review Board, Wuhan, China approved all procedures, which were performed in accordance with the principles of the Declaration of Helsinki.

### Cell Culture and Viral Infection

Huh7.5.1, A549, THP-1^PMA^, Vero, BHK21 and HEK293T cells were purchased from the China Center for Type Culture Collection (CCTCC). The cells were cultured in DMEM (Gibco-Invitrogen) supplemented with 10% FBS (Gibco-Invitrogen) and 1% penicillin/streptomycin. Primary human hepatocytes (PHHs) were purchased from BioIVT (F00995-P) company and cultured in InVitroGRO HI medium. Cells were maintained at 37 °C in a humidified incubator with 5% CO_2_.

The HCV genotype 2a strain JFH1 virus and the J399EM virus were kindly provided by Professor Xinwen Chen (Wuhan Institute of Virology, CAS). To generate the stored viral stocks, the original *in vitro* transcribed hepatitis C virus was inoculated into naïve Huh7.5.1 cells at a multiplicity of infection of 0.1 in DMEM. The infected cells were passaged after the cells reached confluency. The supernatant was collected 8 days after infection and stored at -80 °C 23. Viral titers were quantified using a diagnostic kit (HCV RNA PCR-Fluorescence Probing, KHB, Shanghai, China). The SFTSV strain WCH was obtained from the China Center for General Virus Culture Collection. All experiments related to SFTSV were performed in a biosafety level 3 (BSL-3) facilities, in accordance with institutional biosafety operating procedures. THP-1^PMA^ or Vero cells were infected with the SFTSV strain WCH at a multiplicity of infection (MOI) of 0.1 for viral RNA or plaque analysis 24. The HSV-1 F strain was used to infect A549 or Vero cells for viral RNA or plaque analysis 25. A SARS-CoV-2 S protein pseudovirus system based on VSV and luciferase activity was provided by Professor Huan Yan (State Key Laboratory of Virology, Wuhan University). SARS-CoV-2 S protein pseudovirions were used to infect HEK293T or BHK21 cells stably expressing human angiotensin converting enzyme 2 (hACE2). The transduction efficiency was measured by quantification of the luciferase activity using a microplate reader 26.

### Immunohistochemistry

Immunohistochemistry experiments were used to visualize the expression of p38 and phosphorylated p38 in human liver tissues with an UltraSensitiveTM SP (Mouse/Rabbit) IHC Kit (MX Biotechnology) according to a previously reported method 27 and were performed by Wuhan Hundred Thousand Degree Biological Technology. Briefly, after deparaffinization, rehydration and antigen retrieval, the sections were treated for 10 min with 3% H_2_O_2_ in methanol and blocked for 10 min with 0.5% goat serum at room temperature. The sections were then incubated at a temperature of 4 °C overnight with rabbit anti-p38, rabbit anti-P-p38 or control antibody rabbit IgG. The primary antibodies were subsequently detected by incubation biotinylated goat anti-rabbit IgG, which was followed by incubation with a streptavidin-biotin-peroxidase complex. Specific binding was visualized by using a DAB Kit (MX Biotechnology). The sections were then lightly counterstained with hematoxylin.

### Antibodies and Reagents

Antibodies against P-p38 MAPK (4511), p38 MAPK (9212), and P-MKK3/6 (12280) were purchased from Cell Signaling Technology (Beverly, MA). Antibodies against the HCV core protein (C7-50) (sc-57800) and TAB1 (B-3) (sc-166138) were purchased from Santa Cruz Biotechnology (Dallas, Texas, USA). Antibodies against HA (66006-2-lg and 51064-2-AP), Flag (20543-1-AP), TAB1 (27566-1-AP) and GAPDH (60004-1-lg) were obtained from ProteinTech Group (Wuhan, China). Alexa Fluor 488 (34106ES60), and Cy3 (33108ES60) were purchased from YEASEN (Shanghai, China). SB203580 (S1076) was purchased from Selleck Chemicals (Houston, TX). Dynabeads® Protein G (10004D) was from Invitrogen (Waltham, Massachusetts).

### Plasmids and Small Interfering RNA

The coding regions of human p38 MAPK were amplified by PCR and the PCR products were digested with BamHI/XhoI and cloned directly into the pCMV-3Tag-8 expression vector to generate pCMV-p38α, pCMV-p38αAF and pCMV-TAB1-Flag. The coding regions of the HCV core protein and human p38 MAPK were digested with EcoRI/XbaI and cloned into the pCS2-Flag/HA expression vector to generate the Flag/HA-tagged HCV core protein and the following the Flag-tagged HCV core protein N-terminal or C-terminal truncation constructs: M1-A191 (WT, amino acid residues 1-191), M1-A152 (N1, amino acid residues 1-152), M1-I123 (N2, amino acid residues 1-123), M1-D111 (N3, amino acid residues 1-111), F24-A191 (C1, amino acid residues 24-191), P38-A191 (C2, amino acid residues 38-191), P58-A191 (C3, amino acid residues 58-191), and the pCMV-Flag-p38α and pCMV-HA-p38α plasmids. The coding regions of the HCV core and HCV core 8A mutant constructs were digested with XhoI/BamHI and cloned into the pEGFP-C1 expression vector to generate pEGFP-core, pEGFP-core-8A and pEGFP-TAB1. All constructs were verified by DNA sequencing. The specific small interfering RNA (siRNA) against p38α (sip38) used in this study was purchased from GenePharma (Shanghai, China). The sip38 siRNA sequences were 5'-GGGCAGAUCUGAACAACAUTT-3' (sip38 sense strand, #1) and #2: 5'-CCGAGGUCUAAAGUAUAUATT-3' (sip38 sense strand, #2). The sequence targeted by the negative control RNAi sequence was 5'-UUCUCCGAACGUGUCACGUTT-3' (siNC). The siRNAs were transfected using PepMuteTM siRNA Transfection Reagent (SignaGen, SL100566) at a final concentration of 20 nM according to the manufacturer's instructions.

### Quantitative Real Time PCR (qRT-PCR)

To measure the RNA levels of the indicated genes, the total intracellular RNA content was extracted from cells using TRIzol reagent (Takara), and the first-strand cDNA was reversed-transcribed by using the RevertAid First Strand cDNA Synthesis Kit (ThermoFisher Scientific). The cDNA was quantitated by qRT-PCR using the Bestar® SybrGreen qPCR master mix reagent (DBI® Bioscience). The data shown represent the relative abundance of the indicated RNA normalized to that of GAPDH. The nucleic acid stains (Super GelRed, no.: S-2001) was purchased from US Everbright Inc. The primer sequences for HCV and GAPDH were previously described 28. The primer sequences for p38α, mitogen-activated protein kinase kinase 3 (MKK3) and mitogen-activated protein kinase kinase 6 (MKK6) were as follows p38α: 5'-GCCCAAGCCCTTGCACAT-3' (forward) and 5'-TGGTGGCACAAAGCTGAT GAC-3' (reverse); MKK3: 5'-CCCCAGTCCAAAGGAAAATCC-3' (forward) and 5'-TCTACCACCCCATAGGCTCC-3' (reverse); MKK6: 5'-ATGTCTCAGTCG AAAGGCAAGA-3' (forward) and 5'-CACCTCGTCCCAGTTCCATT-3' (reverse). The titers of the HCV viral supernatant were quantified using a Diagnostic Kit for Quantification of Hepatitis C Virus RNA (PCR-Fluorescence Probing) (KHB Company). All qRT-PCR experiments were performed on an ABI 7500 system according to the manufacturer's instructions.

### Coimmunoprecipitation and Western Blotting

Treated and un-treated cells were collected, and the cell extracts were prepared by incubating the cells in NHG buffer (1% Triton X-100, 10% glycerol, 50 mM HEPES, pH 7.2, and 150 mM NaCl) on ice for 30 min with protease and phosphatase inhibitors (MCE). Cell debris was removed by centrifuging the cell extracts for 15 min at 12000 rpm, and the concentration of total protein was measured using the Pierce™ BCA Protein Assay Kit (Invitrogen). The cleared cell lysates were mixed with specific antibodies for 1 h at room temperature and then coincubated with Dynabeads® Protein G at 4 °C overnight. Nonspecifically bound proteins were removed by washing the beads five times with washing buffer (1% Triton X-100, 10% glycerol, 50 mM HEPES, pH 7.2, and 400 mM NaCl). Equal amounts of proteins were separated by 10% SDS-PAGE and transferred to nitrocellulose membranes (NC membrane, Millipore). The membrane was blocked by incubation with 5% skim milk for 2 h at room temperature. Then, the NC membrane was incubated with the primary antibody at 4 °C overnight, which was followed by incubation with the secondary antibody. Subsequently, the proteins were detected by chemiluminescence using a WesternBrightTM ECL western blotting detection kit (Advansta).

### Confocal Microscopy

Cells were washed three times with PBS, fixed with precooled 4% paraformaldehyde for 15 min at room temperature, permeabilized for 10 min with 0.1% Triton X-100, and blocked for 30 min with 5% bovine serum albumin (BSA) in PBS. Next, the cells were incubated with primary antibody against the specific protein at 4 °C overnight, which was followed by incubation with Alexa Fluor-labeled secondary antibody (Molecular Probes) for 2 h at room temperature. Cell nuclei were subsequently stained with Fluormount with DAPI (antGene). The fluorescence was observed by a confocal laser-scanning microscope (Leica TCS SP2). EGFP and Alexa 488 were excited at a wavelength of 488 nm, Cy3 was excited at 561 nm, and DAPI was excited at 408 nm.

### HCV Titration

To determine the extracellular and intracellular infectious virus titers, Huh7.5.1 cells were infected with the HCV JFH1 or J399EM strain for 2 d, harvested and then treated with SB203580 (a specific p38 inhibitor) for 0, 6 h, 18 h or 24 h before collection. Supernatants containing extracellular virus were harvested to inoculate naïve Huh7.5.1 cells. The infected cells were washed by PBS to remove residual extracellular virus. The cells were scraped with PBS and centrifuged at 2000 g for 5 min. The cell pellets were resuspended in basic DMEM and subjected to three rounds of freeze-thawing to release the intracellular virus particles. The released intracellular virus was centrifuged to remove cell debris. The titers of the extracellular virus and intracellular virus were measured by a modified end-point dilution assay 29. Briefly, 10-fold gradient diluted virus samples were used to infect naïve Huh7.5.1 cells in 96-well plates. The inoculum was incubated with cells for 4 h at 37 °C and then supplemented with fresh complete DMEM. The level of HCV infection was determined 72 h postinfection and calculated by counting EGFP-positive wells under a fluorescence microscope. The viral titer is expressed as the number of focus-forming units per milliliter of supernatant (ffu/ml), which was determined according to the average number of EGFP-positive foci detected at the highest dilutions.

PHHs were thawed in a 37 °C water bath, and 7×10^5^ viable cells were incubated in each well of collagen-coated 12-well culture plates with prewarmed InVitroGRO CP Medium for 24 h. The InVitroGRO HI complete medium was exchanged every 2 days. After 2 days, the cells were infected with JFH1 at an MOI of 5 and incubated with or without drugs. After 72 h, the cells were harvested and analyzed by western blotting and qRT-PCR.

### Prokaryotic Protein Expression and Purification

The cDNA sequence of the HCV core protein was inserted into the expression vector pGEX-6p-1 with the restriction enzymes EcoRI and XhoI. Then, the recombinant vector was transformed into the protein expression bacteria *E. coli* BL21(DE3). *E. coli* BL21(DE3) containing recombinant vector was cultured in LB medium containing 100 µg/ml ampicillin at 37 °C, and 1 mM isopropyl β-D-thiogalactoside (IPTG) was added to induce the synthesis of the fusion protein at 25 °C overnight when the OD was 0.3 at 630 nm. Then, the cells were lysed by ultrasonication at 400 Hz for 125 cycles, and the extract was clarified by centrifugation at 12,000 rpm for 15 min. The GST-core fusion protein in the supernatant was purified by GST-agarose and then desalted by dialysis with 1× enterokinase buffer for 3 h, and then the protein was subjected to ultrafiltration and freeze-dried prior to storage. The protein was analyzed by western blotting.

The cDNA sequence of human p38α was inserted into the expression plasmid pET-28a with the restriction enzymes KpnI and XhoI. Then, the recombinant vector was transformed into the protein expression bacteria *E. coli* BL21(DE3). *E. coli* BL21(DE3) containing recombinant vector was cultured in LB medium with 100 µg/ml kanamycin at 37 °C, and 1 mM IPTG was added to induce the synthesis of the fusion protein at 37 °C for 4 h when the OD was 0.3 at 630 nm. The other processes were the same as the above described protocols for purification of His-p38α protein.

### GST Pulldown

The purified 1 μg His-p38α fusion protein was incubated with Ni-NTA beads in 300 μl binding buffer (20 mM imidazole, 10% glycerol, and 500 mM NaCl, pH 7.9) containing protease inhibitor for 1 h at 4 °C. Then, the supernatant was removed, and the Ni-NTA beads were washed four times with binding buffer. Then, 1 μg GST-core fusion protein was added and incubated at 4 °C overnight. The Ni-NTA beads were washed four times at room temperature for 5 min and then the Ni-NTA beads were resuspended in 20 μl loading buffer and heated at 95 °C for 15 min. The protein was analyzed by western blotting.

### *In vitro* Transcription

The WT and mutant pJFH1 plasmids were digested with the restriction enzyme XbaI and mung bean nuclease (New England Biolabs) 30. The digested plasmids were purified by phenol-chloroform extraction and were used as templates for RNA synthesis, and HCV RNA was synthesized *in vitro* using the MEGAscriptTM T7 Kit (Ambion). The *in vitro* transcripts were treated with DNase I (Promega) at 37 °C for 15 min, and then the purified RNA was obtained using the same method. Equal amounts of purified HCV RNA were electrotransferred into Huh7.5.1 cells to obtain infectious HCV virus, and then the supernatant was collected and stored at -80 °C.

### CRISPR/Cas9

The CRISPR/Cas9 technique was used to obtain p38α or TAB1 knockout Huh7.5.1 cells. The knockout monoclonal Huh7.5.1 cells were not isolated, and p38α or TAB1 knockdown (KD) Huh7.5.1 cells were used in the experiment. The pGL3-U6-sgRNA-PGK-puromycin and pST1374-NLS-flag-linker Cas9 plasmids were used to generate p38α-KD and TAB1-KD cells, respectively. The gRNA targets of human MAPK14 (p38α) (5'-AGCTCC TGCCGGTAGAACGTGGG-3') and human TAB1 (5'-CTGGCCCAGCAGGAG CTCTG-3') were designed with the online software from the Feng Zhang laboratory (http://crispr.mit.edu/). The constructed gRNAs were transfected into Huh7.5.1 cells for 48 h, and then the cells were incubated with puromycin (2 μg/ml) and blasticidin (BSD) (20 μg/ml). After 8 days, the cells were detected by western blotting.

### *In vitro* Kinase Assay and Mass Spectrometry

After transfecting 4 μg Flag-p38α plasmid into HEK293T cells for 24 h, cell lysates were prepared for immunoprecipitation with anti-Flag antibody and Dynabeads® Protein G to obtain the purified Flag-p38α protein. Then, 10 μg of expressed GST-core protein was added and incubated at 30 °C for 1 h in kinase reaction buffer (25 mM Tris-HCl pH 7.5, 0.01% Triton X-100, 10 mM MgCl_2_, 0.5 mM Na_3_VO_4_, 2.5 mM DTT, 0.5 mM EGTA, 0.1 ng/ml BSA, and 50 μM ATP) 31. The reactions were terminated with 5 x SDS and detected by SDS-PAGE. The samples were analyzed by WB with anti-HCV-core and anti-p38 antibodies, or the bands of all samples were analyzed by liquid chromatography-tandem mass spectrometry (LC-MS/MS). The final mass spectrometry detection and analysis results were provided by the Protein Research Technology Center of Tsinghua University.

### Plaque Assay

Cells were seeded in a 12-well plate. When the density of the cultured cells reached 90%, serially diluted virus was added to the cells. After 1 h, the excess virus was removed by washing; MEM containing 2% FBS and 1.5% carboxymethyl cellulose was added to cover the cells (2 ml/well), and the cells were placed in a cell incubator for a suitable number of days. The MEM layer was removed, and the cells were washed three times with PBS. Then, 1 ml of a mixture of 1% crystal violet solution and 10% methanol aqueous solution was added for fixation and staining. After 2 h, the cells were washed with water, and the plaques were counted. The viral infection titer was expressed as pfu/ml.

### Quantification and Statistical Analysis

Graphical representation and statistical analyses were performed using Adobe Photoshop CS6, ImageJ and GraphPad Prism6. Statistical data representative of at least three independent experiments are presented as the means ± SD. The statistical significance was tested using an unpaired, two-tailed Student's *t*-test. Statistical significance was set at *P* < 0.05 (*< 0.05, **< 0.01, and ***< 0.001).

## Results

### The phosphorylation level of p38 is increased in both HCV-infected patient tissues and PHHs

**Figure 1:**
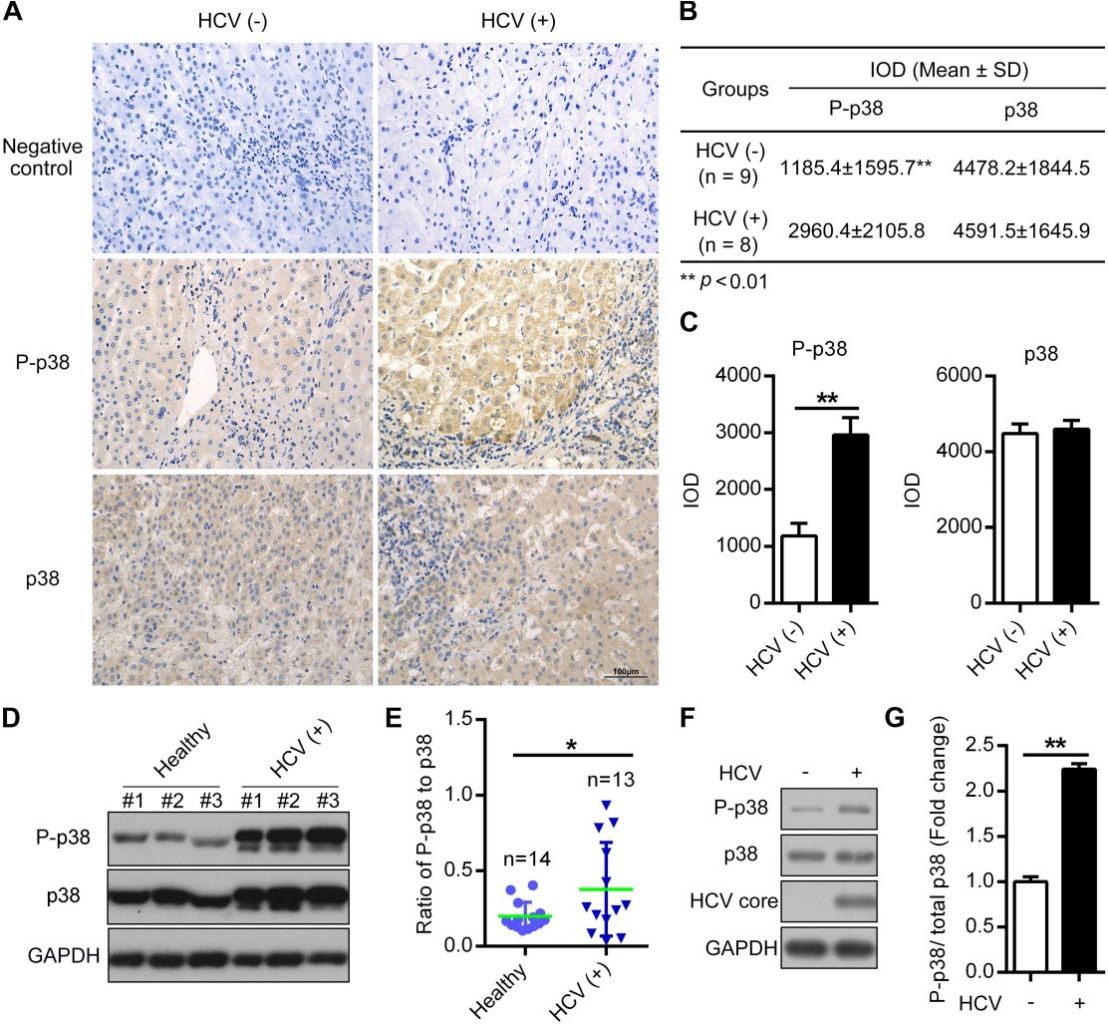
** The phosphorylation level of p38 is increased in both HCV-infected patient tissues and PHHs. (A-C)** Upregulation of the phosphorylated p38 (P-p38) in HCV-infected human liver tissues. Liver cancer tissue sections from HCV-infected (HCV (+)) and HCV-uninfected (HCV (-)) patients were stained with anti-p38 and anti-P-p38 antibodies. IgG antibody was used as the primary antibody in the negative control. (A). Relative expression levels of phosphorylated p38 and total p38 in the liver tissues of HCV (-) and HCV (+) patients were quantitatively analyzed by Image-Pro Plus v6.0 software (B), and the statistics were shown (C). **(D, E)** Upregulation of P-p38 in PBMCs isolated from HCV-infected (HCV (+)) patients. PBMCs were isolated from representative HCV-infected patients and healthy donors. The phosphorylation of p38 (P-p38) and total p38 were detected by western blotting (D), and the ratio of phosphorylated p38 relative to total p38 was quantified by ImageJ software (E). **(F, G)** Upregulation of P-p38 in HCV-infected PHHs. PHHs were infected with JFH1 and the levels of total and phosphorylated p38 were then detected by western blotting (F). The fold change in phosphorylated p38 level relative to the total p38 level was quantified by ImageJ software (G).

HCV infection triggers the activation of multiple signal transduction pathways 32, 33. To explore the potential correlation between HCV infection and p38 activation, we measured p38 activation in hepatocellular carcinoma (HCC) samples by immunohistochemistry (IHC). We found a significant increase in p38 phosphorylation in HCC samples from HCV (+) patients compared with samples from HCV (-) patients, whereas the total p38 protein level was not altered (Figure 1A-C). Furthermore, the phosphorylation of p38 was partially upregulated in peripheral blood mononuclear cells (PBMCs) derived from HCV-infected patients compared with that in PBMCs from healthy donors (Figure 1D-E). In addition, we also detected p38 activation in HCV-infected PHHs. The results showed a similar increase in p38 phosphorylation in HCV-infected PHHs compared to that in HCV-uninfected PHHs (Figure 1F-G). Altogether, these data indicate that p38 activation is associated with HCV infection in both clinical patients and PHHs. P38 activation may play a role in HCV infection.

### HCV infection activates p38α by triggering the interaction of p38α and TAB1

**Figure 2:**
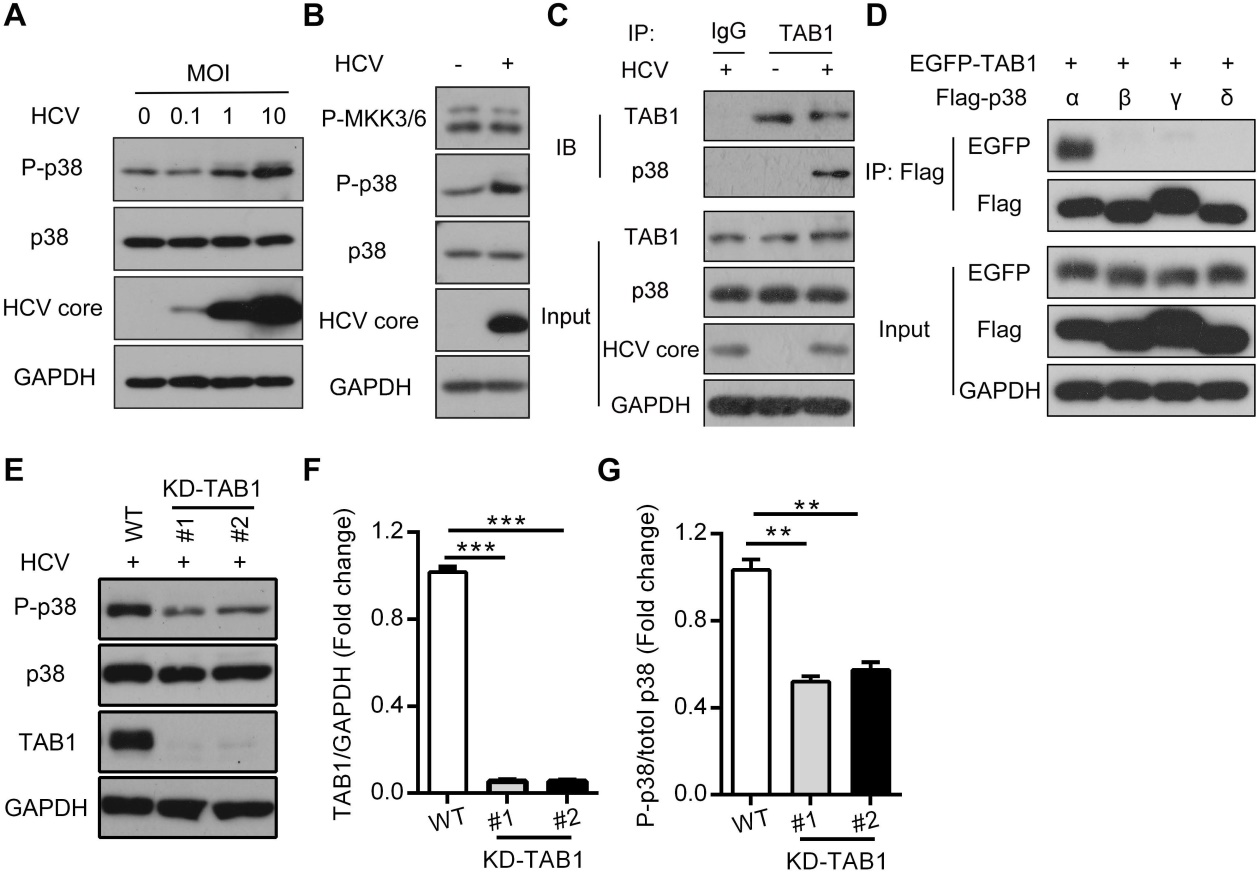
** HCV infection activates p38α by triggering the TAB1-p38α interaction. (A)** Activation effect of HCV infection on p38 in Huh7.5.1 cells. Huh7.5.1 cells were infected with the HCV strain JFH1 at MOIs of 0, 0.1, 1 and 10 for 72 h, and then the total intracellular p38, P-p38 and HCV core protein levels were detected by western blotting. **(B)** MKK3/6-independent activation of p38 in HCV-infected Huh7.5.1 cells. Huh7.5.1 cells were infected (+) or uninfected (-) with the HCV JFH1 strain. P-p38 and P-MKK3/6 were detected by western blotting. **(C)** TAB1-p38 interaction in HCV-infected Huh7.5.1 cells. Huh7.5.1 cells were infected (+) or uninfected (-) with JFH1, and the cell lysates were collected for IP with control antibody (IgG) or TAB1 antibody followed by western blotting analysis of proteins as indicated. **(D)** Interaction between TAB1 and p38α. Cell lysates from HEK293T cells overexpressing Flag-p38α/β/γ/δ, and EGFP-TAB1 were subjected to co-IP with anti-Flag antibody, followed by immunoblotting with anti-EGFP and anti-Flag antibodies. **(E-G)** Effect of TAB1 knockdown on HCV infection-induced p38 activation in Huh7.5.1 cells. TAB1 was knocked down by CRISPR/Cas9 (KD-TAB1) in Huh7.5.1 cells. WT and KD-TAB1 cells were infected with JFH1. P-p38, p38, TAB1 and GAPDH were analyzed by western blotting (E), the fold change of the TAB1 level relative to the GAPDH level was quantified by ImageJ software (F), and the fold change in the phosphorylated p38 level relative to the total p38 level was quantified by ImageJ software (G).

By using the Huh7.5.1 cell model, we confirmed that HCV infection induced p38 phosphorylation in time-dependent (Figure S1) and MOI-dependent manners, while total p38 at both the mRNA and protein levels did not change during viral infection (Figure 2A and Figure S2A). Since MKK3/6 or TAB1 mediated p38 activation 19, we further tested which upstream kinases of p38 were activated and could be potentially responsible for p38 activation. MKK3/6 phosphorylation was not changed by HCV infection (Figure 2B), neither was the total level of MKK3/6 protein or the mRNA level (Figure S2B-C). Instead, we found that HCV infection triggered the interaction of p38, especially p38α but not p38β, p38γ or p38δ, with TAB1 (Figure 2C-D), which is known to cause the autophosphorylation of p38 19. Furthermore, TAB1 knockdown by CRISPR/Cas9 impaired HCV infection-induced p38 phosphorylation (Figure 2E-G and Figure S3), suggesting that TAB1 contributes to HCV infection-induced p38α activation.

### TAB1-mediated p38α activation facilitates HCV replication

**Figure 3:**
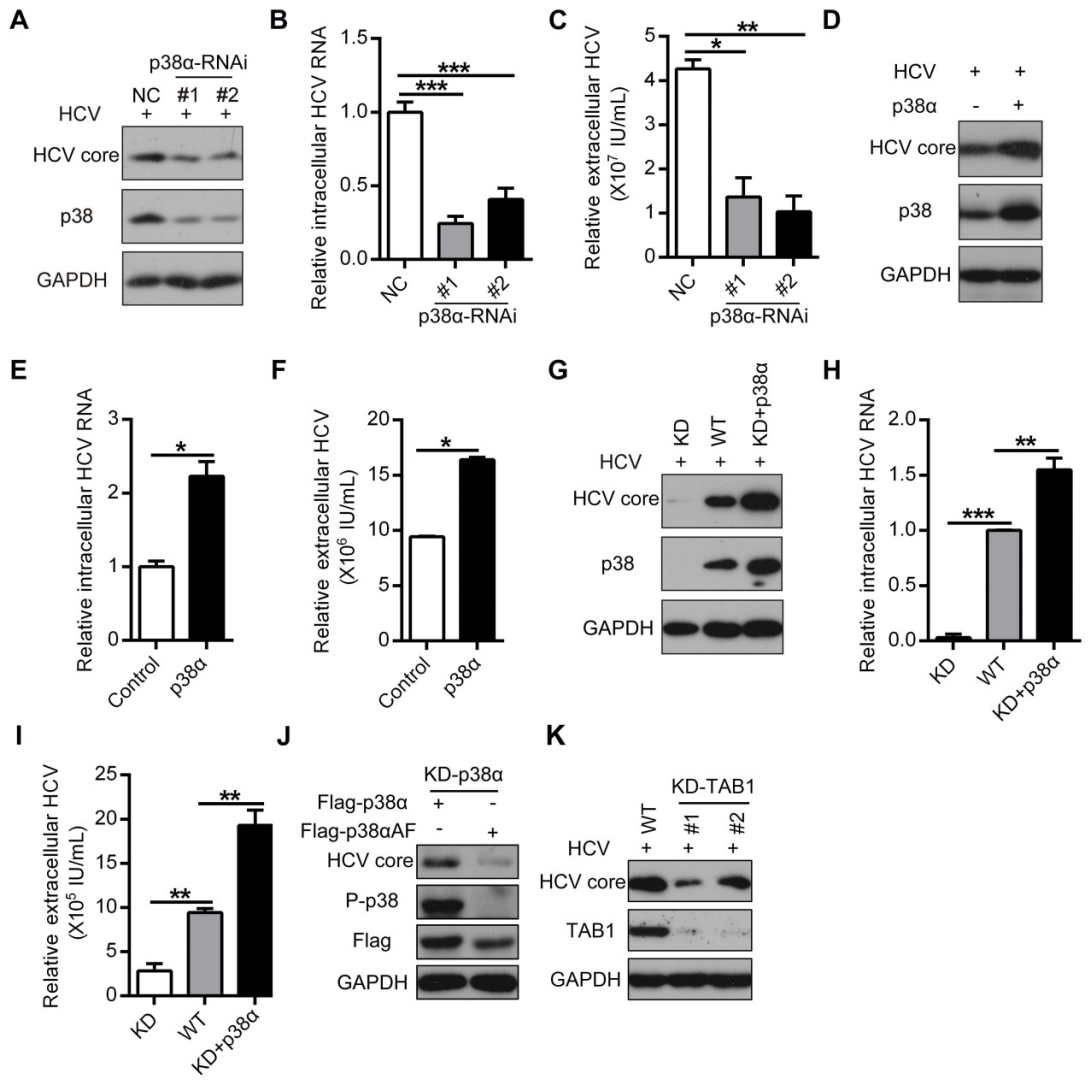
** TAB1-mediated p38α activation facilitates for HCV replication. (A-C)** Inhibitory effect of p38α knockdown on HCV replication in Huh7.5.1 cells. Huh7.5.1 cells were transfected with control siRNA (NC) or siRNA specific for p38 (p38-RNAi #1 and #2) and infected with the HCV JFH1 strain. The expression levels of p38, P-p38, and the HCV core protein were determined by western blotting (A), and the intracellular (B) and extracellular (C) HCV RNA levels were determined by qRT-PCR. **(D-F)** Promotion effect of p38α overexpression on HCV replication in Huh7.5.1 cells. Experiments were performed as described above, except the cells were transfected with the control or p38α expression plasmid. **(G-I)** Effect of p38α on the rescue of HCV replication in p38α-knockdown Huh7.5.1 cells. P38α was knocked down (KD) using CRISPR/Cas9 and p38α was reintroduced into p38α-knockdown cells (KD+p38α). The HCV core protein, p38 and GAPDH were measured by western blotting (G). The intracellular (H) and extracellular (I) HCV RNA levels were measured by qRT-PCR. **(J)** Abolishing effect of p38α activation site mutation on the promotion of p38α to HCV replication. Flag-p38α and Flag-p38αAF plasmids were expressed in p38α-knockdown Huh7.5.1 cells, and HCV infection was detected. **(K)** Impaired effect of TAB1 knockdown on HCV replication in Huh7.5.1 cells. WT and KD-TAB1 cells were infected with JFH1, and the HCV core protein was detected by western blotting.

To further elucidate the role of p38α activation in HCV infection, we knocked down p38α by siRNA (Figure 3A). P38α knockdown significantly decreased HCV replication, as evidenced by the reduction in HCV core protein as well as intracellular and extracellular HCV RNA (Figure 3A-C). In contrast, p38α overexpression enhanced HCV replication (Figure 3D-F). To further verify the specific effect of p38α on HCV infection, p38α knockdown cells (KD) were generated using the CRISPR/Cas9 technique. Again, p38α knockdown significantly reduced HCV core protein and intracellular and extracellular HCV RNA levels compared with those in WT cells (Figure 3G-I). Notably, reintroduction of WT p38α into KD cells restored susceptibility to HCV infection (Figure 3G-I). In sharp contrast, p38αΑF as a mutant form of p38α without a phosphorylation site, failed to rescue HCV core protein expression in p38α knockdown cells (Figure 3J). Consistently, TAB1 knockdown mimicked the effect of p38α knockdown and significantly reduced the HCV core protein expression level compared with that in WT cells (Figure 3K). These results demonstrate that TAB1-mediated p38α activation facilitates HCV replication.

### Pharmaceutical inhibition of p38α suppresses HCV replication

**Figure 4:**
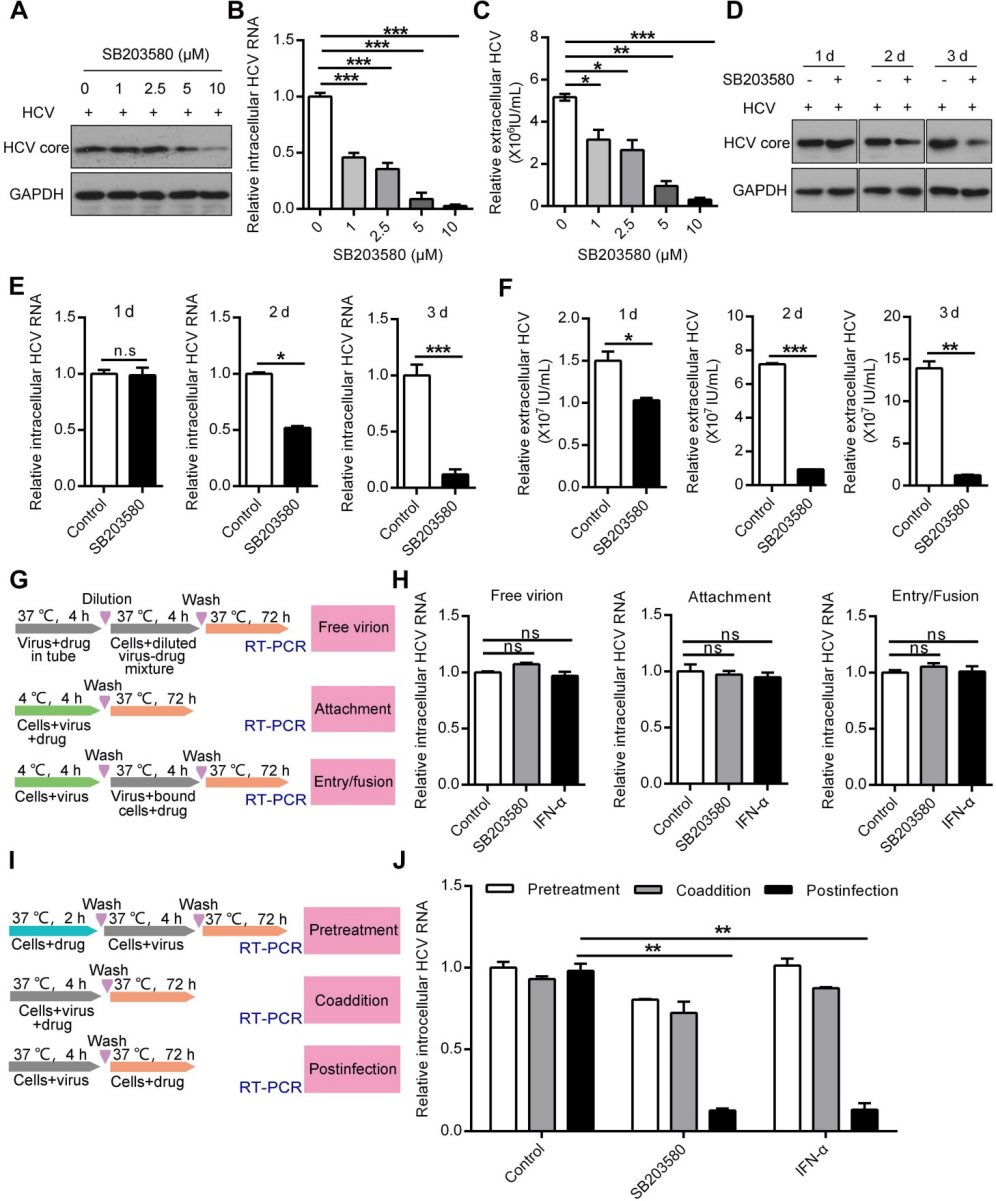
** Pharmaceutical inhibition of p38α suppresses HCV replication at the postinfection stage. (A-C)** Dose-dependent inhibition of p38 by SB203580 suppressed HCV replication, as measured by the HCV core protein (A), the intracellular HCV RNA level (B) and extracellular HCV RNA level (C). Huh7.5.1 cells were preincubated with different doses of the p38 kinase inhibitor SB203580 and infected with JHF1 and HCV replication was detected on day 3. **(D-F)** Time-dependent inhibition of p38 by SB203580 suppressed HCV replication, as measured by the HCV core protein (D), intracellular HCV RNA level (E) and extracellular HCV RNA level (F). Huh7.5.1 cells were preincubated with the p38 kinase inhibitor SB203580 (10 µM) and infected with JHF1. HCV replication was detected on days 1, 2, and 3 after HCV infection. **(G)** A schematic of different SB203580 treatments administered to HCV and host cells. Treatment with SB203580 in different modes was used to study its effect on free viral particles, viral attachment, and viral entry/fusion. **(H)** SB203580 treatment of free virions and SB203580 treatment during attachment and viral entry/fusion in the early stage of the HCV life cycle did not disrupt HCV replication. Huh7.5.1 cells were treated with SB203580 (10 µM) or INF-α (1 µg/µl) as described in (G) and infected with JFH1. Intracellular HCV RNA was measured by qRT-PCR.** (I)** A schematic of different SB203580 treatments administered to HCV host cells. Treatment with SB203580 in different modes was used to study its effect on pretreatment, coaddition, and the postinfection stage. **(J)** SB203580 treatment post infection repressed HCV replication. Huh7.5.1 cells were treated with SB203580 (10 µM) or IFN-α (1 µg/µl) as described in (I) and infected with JFH1. The expression level of intracellular HCV RNA was measured by qRT-PCR.

HCV infection can be inhibited at different stages 34. We took advantage of SB203580 as a specific inhibitor of p38 to modulate p38 MAPK signaling and to determine at which stage p38 may exert its effects. SB203580 inhibits p38 by binding to its ATP pocket but does not affect its phosphorylation by other kinases. SB203580 significantly reduced the expression of the intracellular HCV core protein, the intracellular HCV RNA level and the extracellular HCV RNA level at increased doses (Figure 4A-C). Interestingly, we observed the inhibitory effect of SB203580 on HCV core protein production and intracellular HCV RNA levels only on day 2 and day 3 but not on day 1 in control (without SB203580) and SB203580-treated cells (Figure 4D-E). The difference in extracellular HCV RNA level in control and SB203580-treated cells on day 1 was mild compared to that on day 2 and day 3 (Figure 4F). These results indicate that inhibiting p38 kinase activity by SB203580 treatment can possibly suppress HCV replication at the late stage of HCV infection.

To further determine in which stage of the HCV life cycle the p38 inhibitor SB203580 acts, we conducted a series of experiments according to the protocol schematically shown in Figure 4G. SB203580 and interferon-α (IFN-α) treatment of free virions at the viral attachment and viral entry stages did not affect HCV replication (Figure 4H). In contrast, SB203580 and IFN-α treatment efficiently impaired intracellular HCV RNA production at the postinfection stage (Figure 4I-J). However, neither SB203580 nor IFN-α pretreatment of cells or coadministration of virus had an effect on HCV replication (Figure 4I-J). These results show that p38 inhibition suppresses HCV replication at the postinfection stage. Pharmaceutical inhibition of p38 activation may be a potential strategy for anti-HCV therapy.

### P38α directly interacts with the N-terminus of the HCV core protein

**Figure 5:**
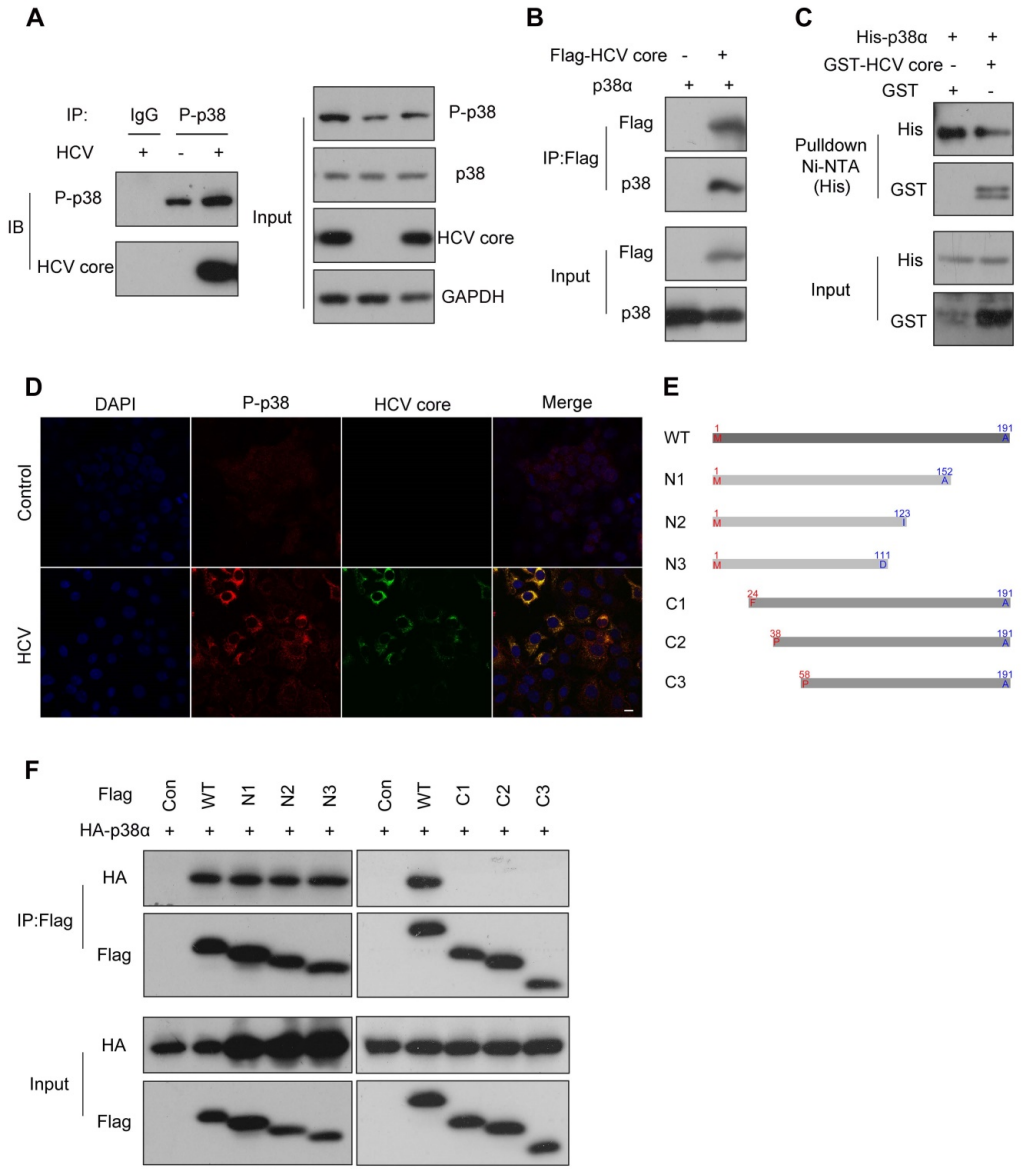
** P38α directly interacts with the N-terminus of the HCV core protein. (A)** The interaction of endogenous phosphorylated p38 with the HCV core protein in Huh7.5.1 cells. Huh7.5.1 cells were infected with JFH1, and cell lysates were prepared for co-IP followed by western blotting. IgG antibody was used for IP of cellular lysates with HCV infection as the control group. P-p38 antibody was used for IP of cellular lysates with or without HCV infection as the experimental group. **(B)** The interaction of exogenous p38α with the HCV core protein in HEK293T cells. HEK293T cells were cotransfected with the Flag-HCV core protein expression plasmid and the p38α expression plasmid and cell lysates were prepared for co-IP followed by western blotting.** (C)** GST pulldown analysis of the direct interaction of p38α and the HCV core proteins. The GST-HCV core and His-p38α proteins were expressed in *E. coli* and purified. The purified proteins were incubated with Ni-NTA beads and the bound proteins were identified by immunoblotting with anti-His and anti-GST antibodies. **(D)** Colocalization of the p38α and HCV core proteins in HCV-infected Huh7.5.1 cells. Cells were fixed in 4% paraformaldehyde, and immunofluorescence staining was performed using an anti-P-p38 antibody (red) and anti-HCV core antibody (green). Cells were counterstained with DAPI to label nuclei (blue). Scale bar, 10 µm. **(E)** Schematic illustration of the full-length HCV core protein (WT) and the N-terminal or C-terminal truncated core protein constructs. WT: 1-191; N1: 1-152; N2: 1-123; N3: 1-111; C1: 24-191; C2: 38-191; C3: 58-191. **(F)** The interaction of p38α with the N- or C-terminal region of the HCV core protein. HEK293T cells were cotransfected with the truncated Flag-core protein expression plasmid and HA-p38α expression plasmid. Cell lysates were prepared for co-IP followed by western blotting of HA and Flag.

As a kinase, p38α is able to phosphorylate many substrates 35. To demonstrate how p38α promotes HCV replication, we immunoprecipitated the endogenous phosphorylated p38 (P-p38) for blotting of potential substrates. We noticed that the HCV core protein was readily detected in the immunocomplex from the experimental group (Figure 5A). Reciprocally, p38α was detected in the immunoprecipitate of the HCV core protein (Figure 5B). To further verify the direct interaction of p38α with the HCV core protein, His-tagged p38α and GST-tagged HCV core proteins expressed in *E. coli* were purified and incubated together with Ni-NTA beads for pulldown assays. Immunoblotting demonstrated that His-tagged p38α captured by Ni-NTA beads also pulled down the GST-tagged HCV core protein (Figure 5C). Immunofluorescence staining further confirmed the colocalization of the P-p38 protein and HCV core protein in the cytoplasm (Figure 5D). To determine the minimum region of the HCV core protein necessary for its interaction with p38α, serial truncations of the HCV core protein were constructed for coimmunoprecipitation (Figure 5E). We found that all C-terminal truncated forms (N1, N2, and N3) effectively coimmunoprecipitated with p38α at a level comparable to that of the full-length HCV core protein. However, N-terminally truncated forms (C1, C2, and C3) completely lost their ability to coimmunoprecipitate (Figure 5F). In particular, the loss of 23 N-terminal amino acid residues (C1) completely abolished the HCV core protein interaction with p38α. These results reveal that p38α directly interacts with the N-terminal region of the HCV core protein, and the HCV core protein may be a potential substrate for p38α.

### P38α phosphorylates the HCV core protein and promotes its oligomerization

**Figure 6:**
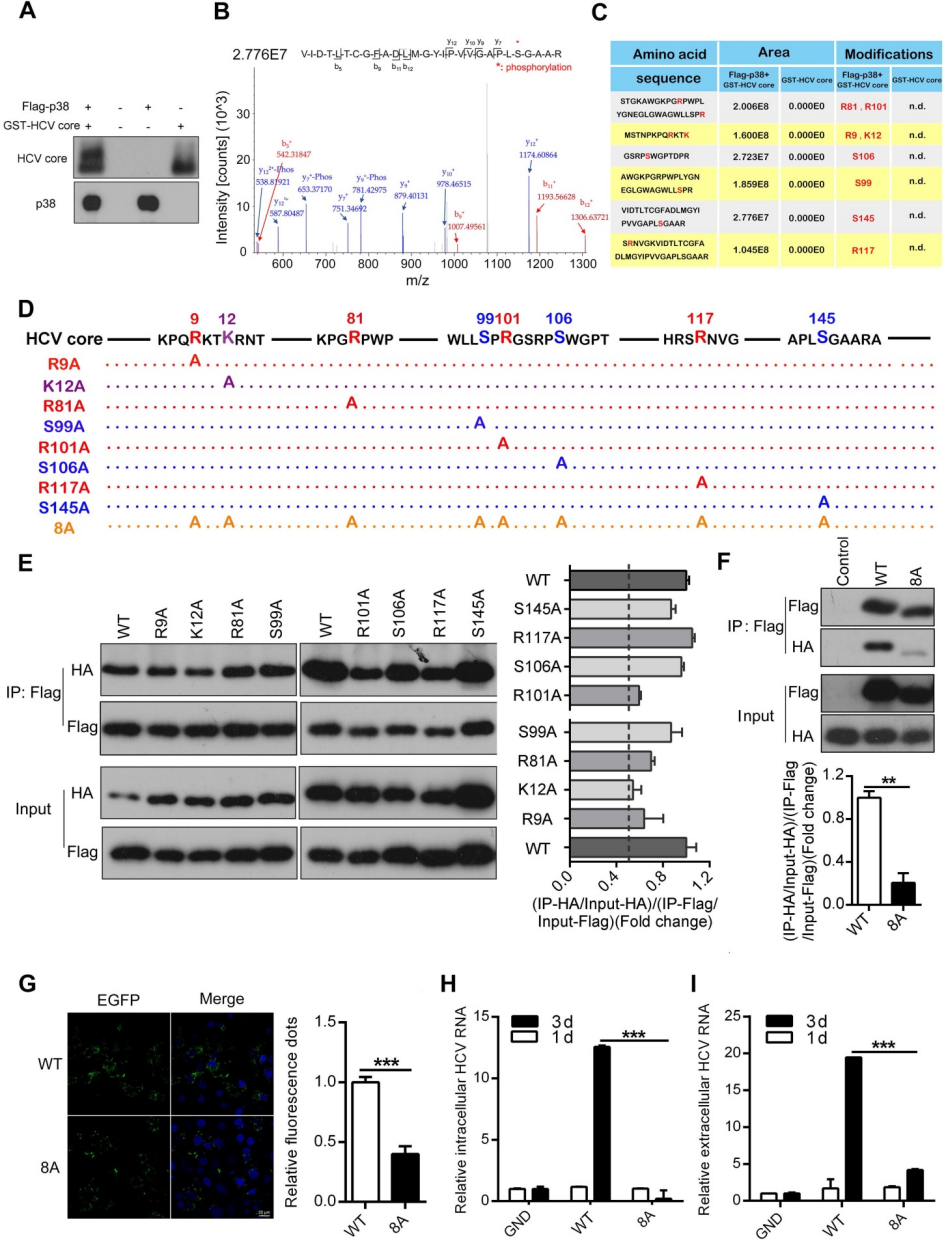
** P38α phosphorylates the HCV core protein and promotes core oligomerization. (A)** *In vitro* phosphorylation of the HCV core protein by p38α. The purified Flag-p38α protein from HEK293T cells was incubated with the purified GST-HCV core protein in kinase reaction buffer. After the reaction, the samples were subjected to western blotting with anti-HCV-core and anti-p38 antibodies. **(B)** Representative mass spectrometry identification of HCV core protein phosphorylation sites catalyzed by p38α. **(C)** Summary and statistics of the sites in the HCV core protein phosphorylated by p38α and their flanking sequences identified by mass spectrometry. Mass spectrometry of the GST-HCV core protein was used as a control. n.d., not detected. **(D)** Illustration of the mutant HCV core protein with p38α-catalyzed phosphorylation sites. **(E)** Impairment by phosphorylation site mutations of HCV core protein oligomerization. HEK293T cells were co-transfected with WT and mutant Flag- and HA-tagged HCV core protein expression plasmids as indicated. Cell lysates were prepared for co-IP (left panel). The fold change in (IP-GST/Input-GST)/(IP-His/Input-His) reflecting the binding efficiency of HCV core protein dimerization was measured by ImageJ software, and the rate for the WT was normalized to 1 (right panel). **(F)** Dimerization defect of the 8A mutant HCV core protein. Experiment was performed as described in (E), and immunoblotting was carried out (top panel). The fold-change analysis was similar to the description of (E). **(G)** Reduced aggregation of 8A mutant HCV core protein in HEK293T cells. EGFP-HCV core-WT or EGFP- HCV core-8A plasmids were transfected into HEK293T cells. EGFP-core-WT and EGFP-core-8A fluorescence aggregation (green) and nuclear fluorescence (blue) were quantified by ImageJ software. **(H, I)** Inhibitory effect of the 8A mutant core protein on HCV replication. Full-length JFH1 RNA containing the GND, WT or 8A mutant HCV core protein sequence was electroporated into Huh7.5.1 cells. The intracellular (H) and extracellular (I) HCV RNA levels on day 1 and day 3 were analyzed by qRT-PCR.

Phosphorylation of the HCV core protein is known to be critical for oligomerization and subsequent virion assembly 36, 37. Therefore, we performed an* in vitro* kinase assay to test whether p38α may phosphorylate the HCV core protein. The purified GST-tagged HCV core protein expressed in *E. coli* was incubated with Flag-p38α immunoprecipitated from transfected HEK293T cells. We noticed a dramatic shift in the band of the HCV core protein incubated with p38α compared to that of the control HCV core protein without incubation with p38α (Figure 6A). The products of the *in vitro* kinase assay were further used for mass spectrometry to analyze the phosphorylation sites of the HCV core protein. A total of eight HCV core protein phosphorylation sites were identified: R9, K12, R81, S99, R101, S106, R117, and S145, suggesting that p38α may phosphorylate the HCV core protein (Figure 6B-C). To investigate the role of phosphorylation sites in oligomerization of the HCV core protein, we mutated each amino acid into alanine (Figure 6D). The WT protein and each mutant HCV core protein were tagged with Flag or HA. Homologous oligomerization of WT and each mutant HCV core protein was measured by coimmunoprecipitation. All mutant HCV core proteins showed slight decreases in oligomerization compared with the WT HCV core protein (Figure 6E). More importantly, oligomerization of the 8A mutant HCV core protein with eight simultaneously mutated phosphorylation sites was significantly reduced (Figure 6F). Furthermore, overexpression of the EGFP-WT HCV core fusion protein caused aggregation that could be quantitated by confocal microscopy. Notably, the EGFP-WT HCV core fusion protein formed many more aggregates than the EGFP-8A mutant HCV core fusion protein (Figure 6G). In addition, p38α knockdown attenuates the dimerization of HCV core protein in Huh7.5.1 cells (Figure S4). These results indicate that phosphorylation of the HCV core protein at these eight sites is important for its oligomerization.

To determine whether the phosphorylation of the HCV core protein affects HCV replication, we incorporated the 8A mutant into the HCV JFH1 genome. The full-length JFH1 RNA genome of the WT, GND (NS5B polymerase-deficient) or HCV core-8A mutant was electroporated into Huh7.5.1 cells. The level of intracellular and extracellular HCV RNA did not substantially change day 1 after electroporation. As a positive control, the amounts of intracellular and extracellular GND HCV RNA were determined to be significantly lower than those of WT HCV RNA (Figure 6H-I) day 3 after infection. Apparently, the amount of intracellular and extracellular 8A mutant HCV RNA were also significantly reduced (Figure 6H-I). These findings indicate that the HCV core-8A mutation significantly attenuates the replication of HCV. Taken together, the results show that the phosphorylation of the HCV core protein catalyzed by p38α is important for the oligomerization of the HCV core protein, which subsequently facilitates HCV replication.

### Disruption of the p38α-HCV core protein interaction affects HCV assembly

**Figure 7:**
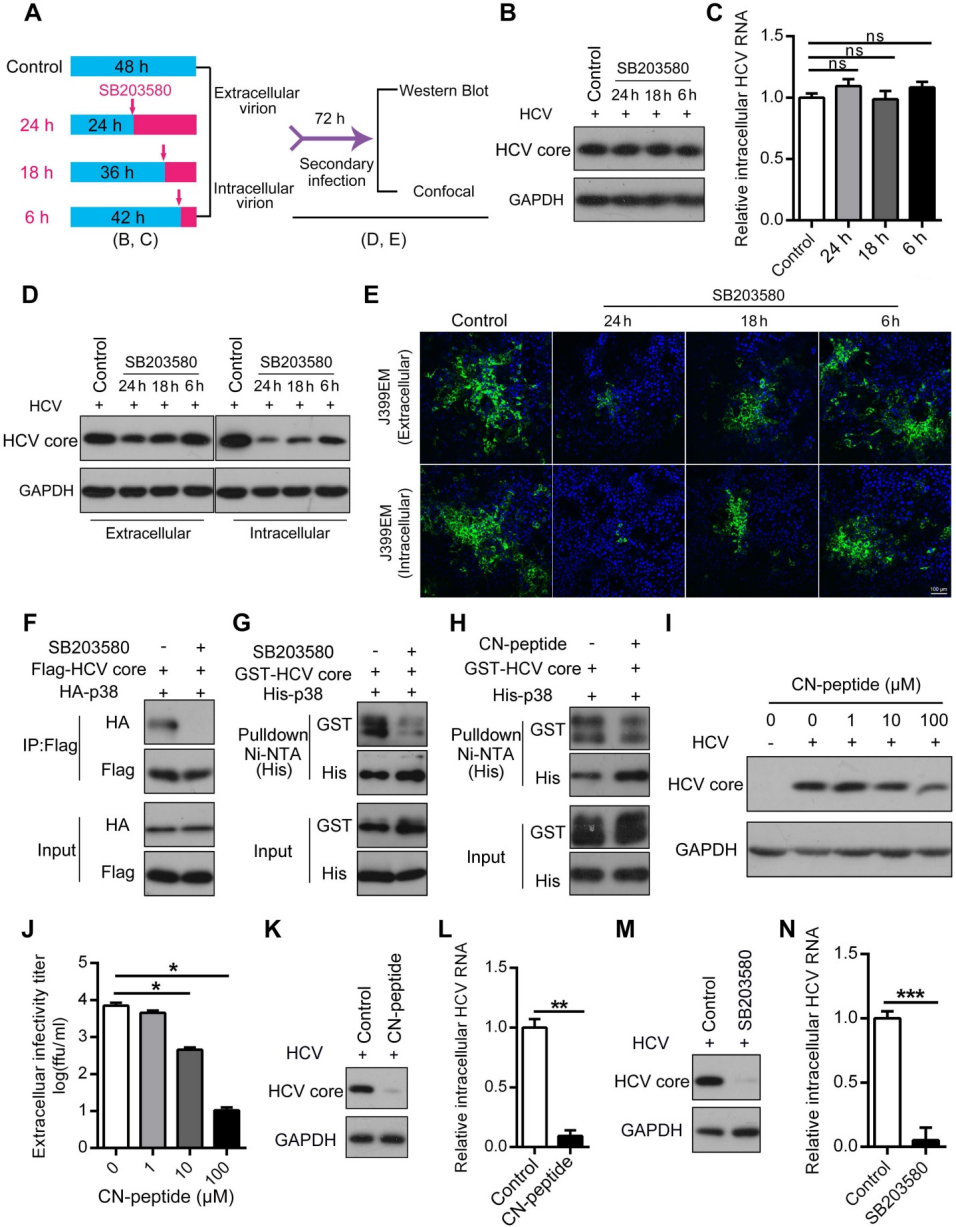
** Disruption of the p38α-core interaction inhibits HCV assembly. (A)** A schematic of HCV single-cycle replication and secondary infection assays. **(B, C)** SB203580 treatment within 24 h (HCV single-cycle) did not affect the biosynthesis of the HCV core protein (B) and RNA (C). Huh7.5.1 cells were infected with J399EM at an MOI of 1, and the cells were then treated with 10 µM SB203580 before collection as described in (A). **(D, E)** Inhibitory effect of SB203580 treatment on HCV assembly according to the HCV secondary infection assay. Huh7.5.1 cells were infected with J399EM at an MOI of 1 and then treated with 10 µM SB203580 before collection as described in (A). Intracellular and extracellular viral particles from the above samples (B) were collected and incubated with naïve cells for 72 h to determine the infectious viral titers. After 72 h, the cellular lysates were identified by immunoblotting (D). The infected cells were subjected to immunofluorescence staining with NS5A (green) and DAPI (blue). The immunofluorescence was examined at 200× magnification (E).** (F-H)** Attenuation effect of SB203580 on the p38α-core interaction. HEK293T cells were preincubated with or without 10 µM SB203580 for 1 h and then transfected with Flag-core and HA-p38α expression plasmids for 24 h. Cell lysates were subjected to co-IP analysis (F). Purified GST-core and His-p38α proteins treated with or without SB203580 (G) and CN-peptide (HCV core N-terminal peptide) (H) were incubated with Ni-NTA beads, and the bound proteins were identified by immunoblotting. **(I, J)** Effect of the inhibition of the CN-peptide on HCV replication in Huh7.5.1 cells. J399EM-infected Huh7.5.1 cells were treated with the CN-peptide for 72 h. The intracellular expression level of the HCV core protein was determined by western blotting (I). The extracellular infectious virus titer was measured by an end-point dilution assay (J). **(K, L)** Inhibitory effect of CN-peptide on HCV replication in PHHs. PHHs were infected with the HCV JFH1 strain and incubated with the CN-peptide for 72 h. The expression level of the HCV core protein was determined by western blotting (K), and the intracellular HCV RNA level was measured by qRT-PCR (L).** (M, N)** Inhibitory effect of SB203580 on HCV replication in PHHs. PHHs were infected with JFH1 at an MOI of 5 and then incubated with 10 µM SB203580 for 72 h. The expression level of the HCV core protein was determined by western blotting (M), and the intracellular HCV RNA level was measured by qRT-PCR (N).

Our evidence indicates that the p38 inhibitor suppressed HCV infection possibly at the assembly stage. To verify this, a single-cycle replication assay, as illustrated in Figure 7A, was performed, and extracellular and intracellular infectious HCV particles were harvested from the single-cycle replication assay for a second round of infection. We showed that SB203580 had no effect on the biosynthesis of the HCV core protein and intracellular HCV RNA within 24 h regardless of SB203580 treatment (Figure 7B-C). However, the amounts of extracellular and intracellular HCV core proteins were significantly decreased in a time-dependent manner (Figure 7D). Notably, SB203580 treatment over time significantly reduced the titer of both the extracellular and intracellular HCV infectious particles (J399EM strain carrying GFP for monitoring virus replication) collected from the single-cycle replication assay (Figure 7E). These observations further indicate that the activation of p38α represses HCV infection possibly at the assembly stage.

**Figure 8:**
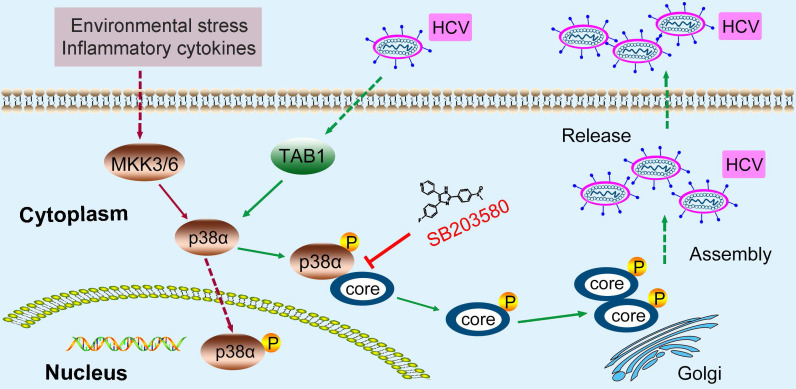
** A model of the novel feedforward mechanism boosting HCV infection.** HCV infection induces p38α autophosphorylation by triggering the interaction of TAB1 and p38α. Activated p38α in turn catalyzes the phosphorylation of the HCV core protein, thereby facilitating core oligomerization and viral assembly.

The ability of SB203580 to bind to the ATP-binding pocket of p38 may not sufficiently explain its specificity for p38α. In addition to suppressing p38α, SB203580 is known to suppress p38β and AKT 38, 39. SB203580 treatment impaired the interaction of p38α and the HCV core protein measured by coimmunoprecipitation (Figure 7F). Similarly, pulldown experiments demonstrated that SB203580 significantly disrupted the direct interaction between p38α and the HCV core protein (Figure 7G). Since the N-terminal region (1-23 amino acid residues) of the HCV core protein mediates its interaction with p38α, we synthesized this region as an N-terminal peptide (CN-peptide) to interfere with the interaction (Figure S5). The *in vitro* pulldown assay demonstrated that CN-peptide impaired the interaction (Figure 7H). Furthermore, CN-peptide inhibited HCV replication in Huh7.5.1 cells in a dose-dependent manner, as measured by the HCV core protein level of p38α and the HCV core protein (Figure 7I). The number of infectious HCV particles in the supernatant was dose-dependently reduced by CN-peptide treatment (Figure 7J). These results suggest that disruption of the p38α-HCV core protein interaction by either the inhibitor SB203580 or CN-peptide, is an effective strategy for anti-HCV therapy. To further examine this, we infected PHHs with HCV and tested the effect of CN-peptide and SB203580 on HCV replication. CN-peptide treatment significantly reduced the level of the HCV core protein and caused a 91% reduction in intracellular HCV RNA in PHHs (Figure 7K-L). Furthermore, striking reductions in the HCV core protein and intracellular HCV RNA were also observed in SB203580-treated PHHs (Figure 7M-N). These findings suggest the potential application of this strategy for anti-HCV therapy. Altogether, our study reveals the role of p38 activation, which is hijacked by HCV and a new feedforward mechanism boosts HCV infection (Figure 8). Disruption of the p38 interaction by the HCV core protein may serve as a new therapeutic strategy against HCV infection.

### The p38 inhibitor SB203580 suppresses SFTSV, HSV-1 and SARS-CoV-2

**Figure 9:**
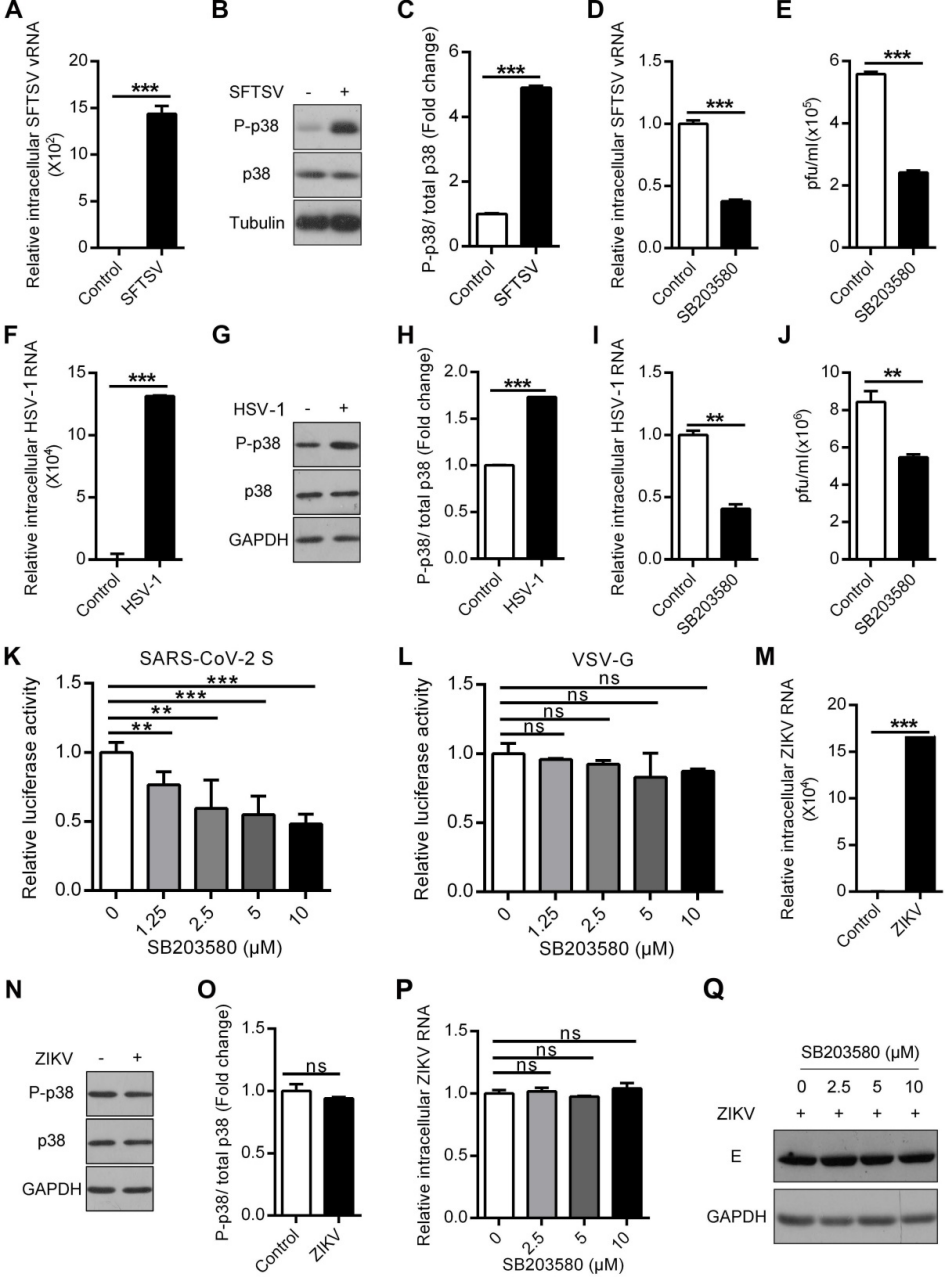
** The p38 inhibitor SB203580 suppresses SFTSV, HSV-1 and SARS-CoV-2. (A-C)** Effect of SFTSV on p38 activation in THP-1^PMA^ cells. The intracellular SFTSV RNA (A) and p38 (B) levels were analyzed in SFTSV-infected THP-1^PMA^ cells. The fold change in the phosphorylated p38 was quantified (C). **(D, E)** Inhibition of SFTSV by SB203580 in THP-1^PMA^ cells. THP-1^PMA^ cells were infected with SFTSV at an MOI of 0.1 and then incubated with SB203580 (10 µM) for 48 h. The intracellular SFTSV RNA level was measured by qRT-PCR (D) and the supernatant viral titer was detected in Vero cells by the plaque assay (E).** (F-H)** Effect of HSV-1 on p38 activation in A549 cells. The intracellular HSV-1 RNA (F) and p38 (G) levels were analyzed in HSV-1-infected A549 cells. The fold change in the phosphorylated p38 was quantified (H). **(I, J)** Suppression of HSV-1 by SB203580 in A549 cells. A549 cells were infected with HSV-1 at an MOI of 1 and then incubated with SB203580 (10 µM) for 48 h. The intracellular HSV-1 RNA level was measured by qRT-PCR (I) and the supernatant viral titer was detected in Vero cells by the plaque assay (J). **(K, L)** Inhibitory effect of SB203580 on SARS-CoV-2 entry into HEK293T/hACE2 cells. HEK293T/hACE2 cells were transduced with SARS-CoV-2 S pseudovirions (K) or VSV G pseudovirions (L) at an MOI of 0.1, and then treated with different concentrations of SB203580 (0, 1.25 µM, 2.5 µM, 5 µM and 10 µM). The intracellular luciferase activity was measured 20 h post incubation. **(M-O)** Insensitivity of p38 activation to ZIKV in A549 cells. The intracellular ZIKV RNA (M) and p38 (N) levels were determined in ZIKV-infected A549 cells. The fold change in phosphorylated p38 was quantified (O).** (P, Q)** The lack of an effect of SB203580 on ZIKV in A549 cells. A549 cells were infected with ZIKV at an MOI of 1 and then incubated with SB203580 for 48 h. The expression levels of intracellular ZIKV RNA (P) and E protein (Q) were determined.

P38 MAPK is an important kinase that is widely expressed in various tissues and cells. P38 is almost always activated in host cells infected by many viruses. We wanted to know whether p38 activation plays a similar role in infections by multiple viruses. An increase in the amount of phosphorylated p38 was shown in SFTSV-infected THP-1^PMA^ cells, but SFTSV infection did not change the expression of total p38 (Figure 9A-C), indicating that SFTSV infection induced p38 activation. Interestingly, treatment with SB203580 obviously reduced the expression of intracellular SFTSV RNA and the number of extracellular virus particles (Figure 9D-E). Similarly, HSV-1 infection was found to significantly activate p38 in A549 cells (Figure 9F-H), and treatment with the p38 inhibitor SB203580 was shown to prevent cellular infection with HSV-1 (Figure 9I-J). Recently, SARS-CoV-2 was revealed to activate many host cell kinases, and inhibition of the activation of this kinase was presented as a promising antiviral strategy 40. We then determined whether SB203580 could inhibit the entry of SARS-CoV-2 S pseudovirions into HEK293T cells stably expressing the hACE2 system. As expected, SB203580 treatment inhibited the entry of SARS-CoV-2 S pseudovirions in a dose-dependent manner (Figure 9K-L). In contrast, p38 MAPK did not respond to Zika virus (ZIKV) infection (Figure 9M-O). The levels of ZIKV RNA and E protein did not change when ZIKV-infected cells were treated with different concentrations of SB203580 (Figure 9P-Q). Many previous studies reported that infections by some viruses, such as HBV, HIV-1 and EBOV, can increase p38 phosphorylation, and SB203580 can also inhibit the replication of the corresponding viruses 10, 12, 41. All these data suggest that there is a common pattern in which multiple viruses, including SFTSV, HSV-1 and SARS-CoV-2, hijack p38 activation to facilitate viral replication.

## Discussion

P38 activation by many viruses, such as HCV 7, influenza virus 8, EV71 9, HIV 10, DENV 11, HBV 12 and EBOV 41, has been reported. However, the underlying mechanism remains unknown and the role of p38 activation in viral infection has never been revealed. In our study, we showed the association of HCV infection with p38 activation in clinical samples. We further dissected the role of p38 activation in HCV infection and the underlying mechanism. We finally demonstrated that virus-hijacked p38 activation is an important event for viral replication and that blockage of p38 activation may be a potential antiviral strategy that could be used against the fatal virus SFTSV, the widespread human virus HSV-1, and the newly emerged lethal virus SARS-CoV-2. Our study revealed several novel points.

First, our study found that HCV infection induced p38 activation by triggering the p38α-TAB1 interaction, independently of the MKK3/6 pathway. In general, p38α is most easily activated by external stimuli through the MKK3/6 classical signaling pathway 35. Alternatively, TAB1 can interact with only p38α and cause its autophosphorylation with limited stimulation. TNF-α or peroxynitrite can trigger the interaction between endogenous TAB1 and p38α in HEK293T cells 19. The amyloidogenic light chain (AL-LC) protein activates p38α by TAB1-mediated p38α autophosphorylation in primary amyloidosis patients, causing oxidative stress, cellular dysfunction and apoptosis 20. To the best of our knowledge, viral infection was first found to activate p38α by the TAB1-dependent signaling pathway.

Second, we identified a feedforward mechanism by which HCV hijacks host p38 MAPK to promote infection. HCV infection induces p38α autophosphorylation by triggering the interaction of TAB1 and p38α. Activated p38α in turn catalyzes the phosphorylation of the HCV core protein, thereby facilitating HCV core oligomerization and viral assembly. Thus, a vicious cycle of HCV infection is established based this feedforward loop. Theoretically, disruption of such a vicious cycle would be effective to combat HCV infection. Although the currently available NS5A inhibitors Sovaldi, Harvoni and Epclusa have reduced side effects following treatment with early NS3/4A protease inhibitors and can treat type 1-6 chronically HCV-infected patients, these drugs still cause side effects, are costly, involve long-term treatment, and target a virus that is prone to drug resistance 2, 3, 42. Therefore, the discovery of additional antiviral drug targets in host cells and the development of successful antiviral treatments remain a challenge. Our study revealed that targeting host p38α by disrupting the interaction of p38α and the HCV core protein is a potential anti-HCV strategy.

Third, we revealed a common pattern in which the virus hijacks p38 activation to facilitate viral replication and that blockage of p38 activation may be a potential antiviral strategy. Previous reports have shown that host cells could respond to infections by some viruses by activating the p38 MAPK signaling pathway and that the p38 inhibitor SB203580 also inhibited corresponding viral replication. Consistently, SFTSV, HSV-1 and SARS-CoV-2 infections could all induce the phosphorylation of p38 in host cells. The p38 inhibitor SB203580 showed significant inhibitory activity against SFTSV, HSV-1 and SARS-CoV-2. It is well known that the tick-borne virus SFTSV causes severe fever with thrombocytopenia syndrome (SFTS), which was listed among the top 10 priority infectious diseases by the World Health Organization (WHO) due to its high fatality rate of 12%-50% and the possibility of pandemic transmission 4. HSV-1 infects approximately 80% of the world's populations, of which approximately 40% suffer from recurrent infection 5. Now, SARS-CoV-2 infection causes a global emergency due to COVID-19. P38 MAPK inhibitors are widely used in drug development research. The p38 MAPK inhibitor losmapimod is a drug candidate for treatment of cardiovascular disease, and its use for treatment of facioscapulohumeral muscular dystrophy (FSHD) has been tested in phrase I and II clinical trials, which showed its safety and tolerability 43, 44. Similar candidate drugs may show potential as antiviral drugs, especially against SFTSV, HSV-1 and SARS-CoV-2.

In summary, HCV infection was found to induce p38α autophosphorylation by triggering the interaction of TAB1 and p38α. And then the activated p38α catalyzes the phosphorylation of the HCV core protein, thereby facilitating HCV assembly and promoting HCV replication. Similar to HCV, SFTSV, HSV-1 or SARS-CoV-2 infection also activates p38 MAPK, and the treatment of the p38 inhibitor has antiviral activities against SFTSV, HSV-1 and SARS-CoV-2. Thus, virus-hijacked p38 activation may be an important event for viral replication and the blockage of virus-hijacked p38 activation may be a potential antiviral strategy.

## Abbreviations

SARS-CoV-2: severe acute respiratory syndrome coronavirus 2
COVID-19: coronavirus disease 2019
DAAs: direct-acting antivirals
GST: glutathione S-transferase
HCC: hepatocellular carcinoma
HCV: hepatitis C virus
HSV-1: herpes simplex virus type 1
IFN-α: interferon-α
IHC: immunohischemistry
MKK3: mitogen-activated protein kinase kinase 3
MKK6: mitogen-activated protein kinase kinase 6
MOI: multiplicity of infection
P38 MAPK: p38 mitogen-activated protein kinase
PBMCs: peripheral blood mononuclear cells
PHHs: primary human hepatocytes
qRT-PCR: quantitative real time PCR
SFTS: severe fever with thrombocytopenia syndrome
SFTSV: severe fever with thrombocytopenia syndrome virus
TAB1: TGF-beta activated kinase 1 (MAP3K7) binding protein 1
TNF-α: tumor necrosis factor-α
ZIKV: zika virus
