# Supplementary material for: Virus-induced p38 MAPK activation facilitates viral infection
Source: Theranostics. 2020 Oct 30;10(26):12223–40. doi: 10.7150/thno.50992 (PMC7667676; doi:10.7150/thno.50992)
Supplement: Supplementary file 1 — Supplementary figures and tables. [file thnov10p12223s1.pdf]

Supplementary Information

Virus-induced p38 MAPK activation facilitates viral infection

Yuting Cheng[1, 2,§], Fang Sun[1,§], Luyao Wang[1], Minjun Gao[1], Youli Xie[3], Yu Sun[4], Huan Liu[3], Yufeng Yuan[3], Wei Yi[5], Zan Huang[1], Huan Yan[1], Ke Peng[6], Yingliang Wu[1], Zhijian Cao[1, 7,¶]

[1] State Key Laboratory of Virology and Modern Virology Research Center, College of Life Sciences, Wuhan University, Wuhan 430072, Hubei, China

[2] Jiangsu Agri-animal Husbandry Vocational College, Jiangsu Key Laboratory for High-Tech Research and Development of Veterinary Biopharmaceuticals, Taizhou, 225300, China

[3] Zhongnan Hospital of Wuhan University, Wuhan 430071, Wuhan 430072, Hubei, China

[4] School of Medicine, Wuhan University of Science and Technology, Wuhan 430065, Hubei, China

[5] Department of Neurosurgery, Renmin Hospital of Wuhan University, Wuhan 430060, Hubei, China

[6] Wuhan Institute of Virology, Chinese Academy of Sciences, Wuhan 430071,

Hubei, China

[7] Hubei Province Engineering and Technology Research, Center for Fluorinated Pharmaceuticals, Wuhan University, Wuhan 430071, Hubei, China

Running title: Pharmacological blockage of p38 activation is a promising antiviral strategy

§ These authors contributed equally.

¶ Correspondence: State Key Laboratory of Virology and Modern Virology Research Center, College of Life Sciences, Wuhan University, Wuhan 430072, Hubei, China. Tel: +86-27-68752831, Fax: +86-27-68756746. E-mail: zjcao@whu.edu.cn (Zhijian Cao).

Supplementary Figure Legends

Figure S1. HCV activates p38 in a time-dependent manner. Huh7.5.1 cells were infected with JFH1 (MOI=1) for 0, 24 h, 48 h and 72 h. Cell lysates were prepared, followed by detecting intracellular total p38, P-p38 and HCV core protein levels by western blotting.

Figure S2. HCV infection has no effect on the mRNA expression of p38α, MKK3 and MKK6. (A) Huh7.5.1 cells were infected with the HCV strain JFH-1 at MOIs of 0, 0.1, 1 and 10 for 72 h, and then intracellular p38α mRNA levels were analyzed via qRT-PCR. **(B, C)** Huh7.5.1 cells were infected (+) or uninfected (-) with the HCV JFH-1 strain at the MOI of 1 for 72 h. The intracellular MKK3 (B) and MKK6 (C) mRNA levels were detected via qRT-PCR.

Figure S3. Impairment of TAB1 knockdown on p38 activation in HCV-uninfected Huh7.5.1 cells. (A) TAB1 was knocked down by CRISPR/Cas9 (KD-TAB1) in Huh7.5.1 cells. The P-p38, p38, TAB1 and GAPDH were analyzed by western blotting. **(B)** The fold change of the TAB1 level relative to the GAPDH level was quantified by ImageJ software. **(C)** The fold change in the phosphorylated p38 level relative to the total p38 level was quantified by ImageJ software.

Figure S4. p38α knockdown attenuates the dimerization of HCV core protein in Huh7.5.1 cells. Wild type and p38α knockdown Huh7.5.1 cells were co-transfected Flag-core and HA-core plasmids, respectively. After 48 h, cell lysates were prepared for co-IP followed by western blot.

Figure S5. Amino acid sequence, purification and identification of CN-peptide. (A) Amino acid sequence of CN-peptide. **(B)** The MS analysis of CN-peptide. **(C)** The HPLC analysis of CN-peptide.

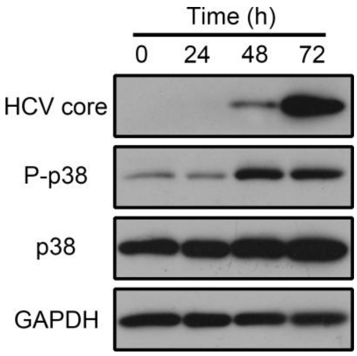

Figure S1

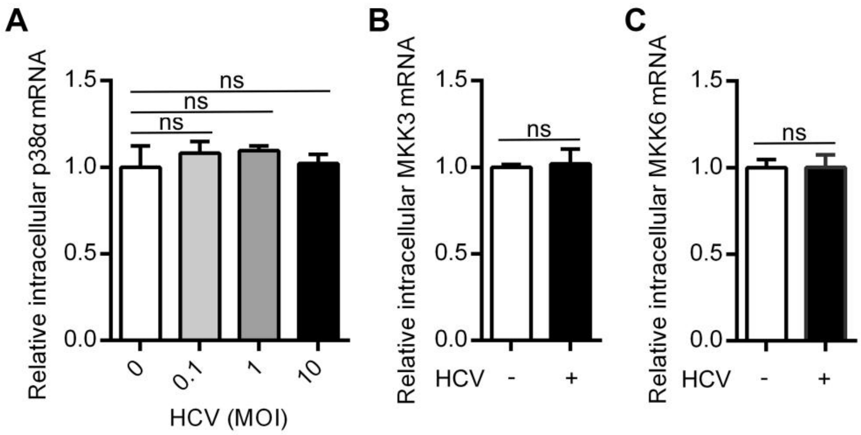
A
Relative intracellular p38α mRNA
1.5
1.0
0.5
0.0
ns
ns
ns
0
0.1
1
10
HCV (MOI)
B
Relative intracellular MKK3 mRNA
ns
HCV
-
+
C
Relative intracellular MKK6 mRNA
ns
HCV
-
+

Figure S2

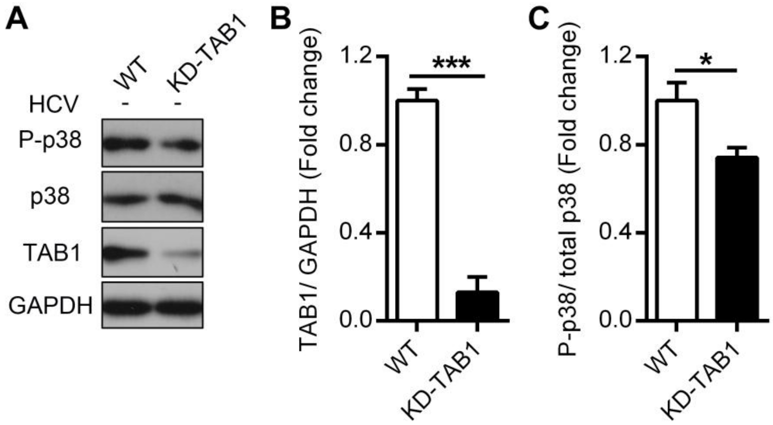
A
WT
KD-TAB1
HCV
-
-
P-p38
p38
TAB1
GAPDH
B
TAB1/ GAPDH (Fold change)
1.2
0.8
0.4
0.0
***
WT
KD-TAB1
C
P-p38/ total p38 (Fold change)
1.2
0.8
0.4
0.0
*
WT
KD-TAB1

Figure S3

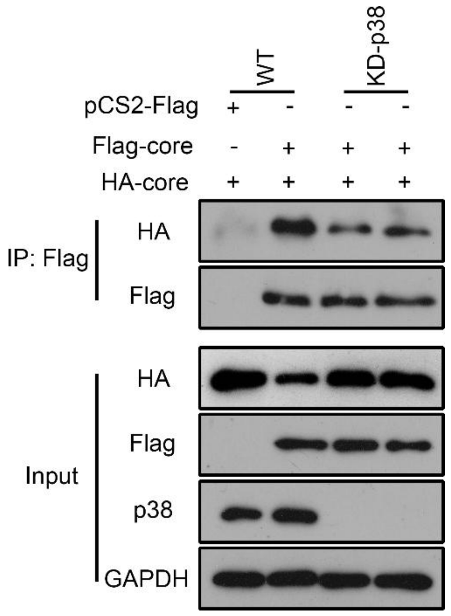
WT
KD-p38
pCS2-Flag + - - -
Flag-core - + + +
HA-core + + + +
IP: Flag
HA
Flag
Input
HA
Flag
p38
GAPDH

Figure S4

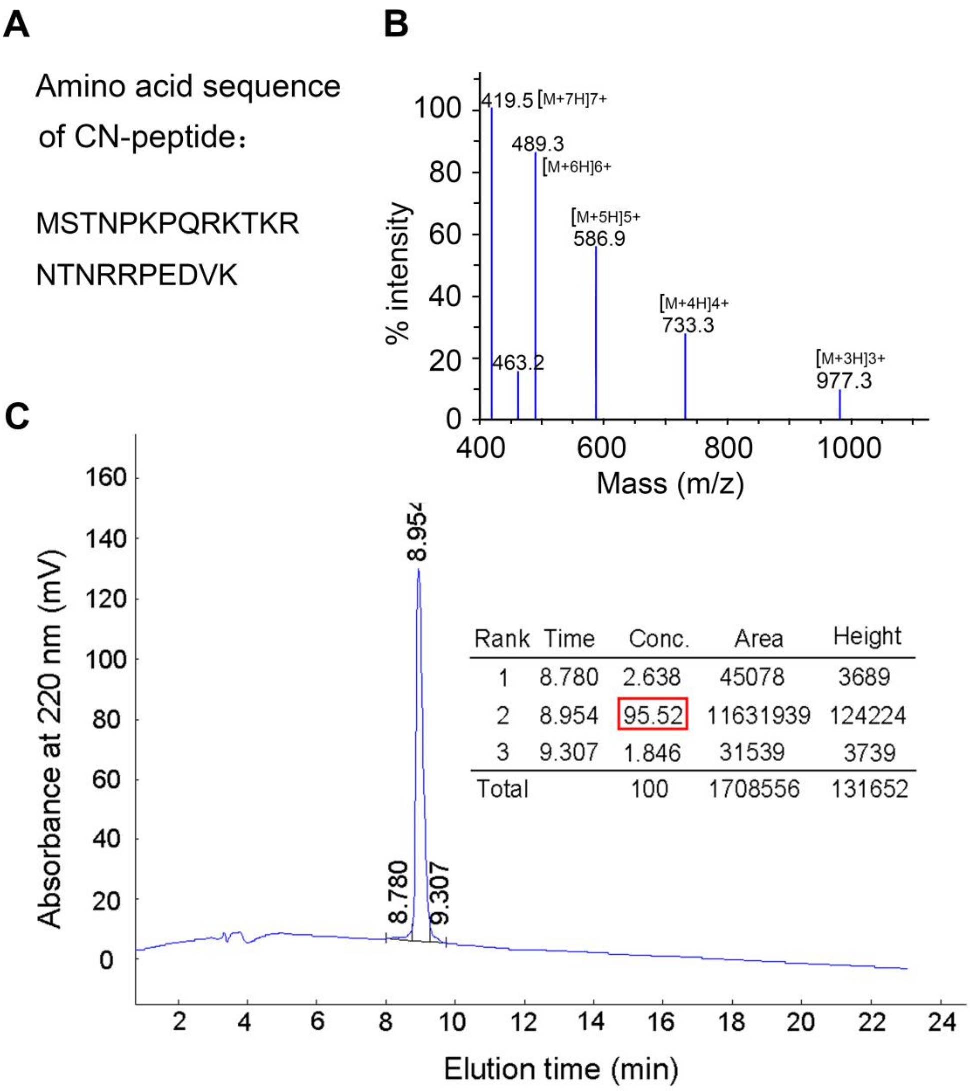
A
Amino acid sequence of CN-peptide：
MSTNPKPQRKTKR
NTNRRPEDVK
B
% intensity
Mass (m/z)
419.5 [M+7H]7+
489.3 [M+6H]6+
[M+5H]5+ 586.9
[M+4H]4+ 733.3
[M+3H]3+ 977.3
463.2
C
Absorbance at 220 nm (mV)
Elution time (min)
8.954
8.780
9.307
Rank Time Conc. Area Height
1 8.780 2.638 45078 3689
2 8.954 95.52 11631939 124224
3 9.307 1.846 31539 3739
Total 100 1708556 131652

Figure S5
